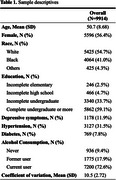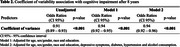# Early Cognitive Dispersion and Risk of Cognitive Impairment: Insights from the ELSA‐Brasil Study

**DOI:** 10.1002/alz70857_100767

**Published:** 2025-12-25

**Authors:** Laiss Bertola, Isabela M Bensenor, Paulo A Lotufo, Claudia Kimie Suemoto

**Affiliations:** ^1^ University of Sao Paulo Medical School, Sao Paulo, Sao Paulo, Brazil; ^2^ University of Sao Paulo Medical School, Sao Paulo, Brazil; ^3^ University of São Paulo Medical School, São Paulo, SP, Brazil; ^4^ University of São Paulo Medical School, São Paulo, São Paulo, Brazil

## Abstract

**Background:**

Neuropsychological assessments predominantly rely on mean‐level performance. However, middle‐aged and older adults may exhibit increased intraindividual cognitive variability (IICV) as an early sign of cognitive impairment, even when classified as cognitively normal. IICV, also seen as dispersion of scores, reflects inconsistencies in cognitive function and has been linked to higher dementia risk. We aimed to investigate whether baseline IICV predicts cognitive impairment over eight years.

**Method:**

We analyzed data from 9,914 ELSA‐Brasil participants, a multicenter, prospective cohort study with three waves, approximately four years apart. Baseline neuropsychological assessments included measures of episodic memory and executive function. Participants with two or more regression corrected‐based norms cognitive scores below ‐1.5 SD were excluded for possible baseline deficits. IICV was measured using the coefficient of variation (CoV), which adjusts for global cognitive ability differences. CoV is calculated by dividing the intraindividual standard deviation by the participant's battery mean score (i.e., the average of the demographic‐adjusted T‐scores for all test variables). CoV scores were scaled (Mean = 10, SD = 3) to enhance clinical interpretation (higher CoV values indicate lower IICV). Cognitive impairment was determined at Wave 3 using education‐adjusted Mini‐Mental State Exam (MMSE) cutoffs. Logistic regression models, adjusted for sociodemographic factors (age, sex/gender, education, race/ethnicity) and clinical confounders (depressive symptoms, hypertension, diabetes, alcohol consumption), were used to estimate odds ratios (ORs) for cognitive impairment.

**Result:**

Participants had a mean age of 50.7 ± 8.7 years; 55% were women, 7% had less than a high school education, and 41% identified as Black/Brown (Table 1). At baseline, 2.4% of the participants had a CoV scaled score of 5 or lower (indicative of atypical IICV). After eight years, 13% of the sample was classified as having cognitive impairment. Logistic regression indicated that for every one‐unit increase in CoV, the odds of developing cognitive impairment decreased by 6% (OR 0.94, CI95% 0.92 – 0.96) (Table 2).

**Conclusion:**

Higher IICV at baseline was associated with an increased risk of cognitive impairment eight years later. These findings underscore the importance of considering cognitive dispersion in neuropsychological assessments once IICV may be a valuable target for identifying individuals at risk for preclinical impairment.